# When to investigate for secondary hyperhidrosis: data from a retrospective cohort of all causes of recurrent sweating

**DOI:** 10.1080/07853890.2022.2102675

**Published:** 2022-07-29

**Authors:** Nived Collercandy, Camille Thorey, Elisabeth Diot, Leslie Grammatico-Guillon, Eve Marie Thillard, Louis Bernard, François Maillot, Adrien Lemaignen

**Affiliations:** a Service de Médecine interne et Maladies infectieuses, Centre Hospitalier Universitaire de Tours, Tours, France; b Service de Médecine interne et Immunologie clinique, Centre Hospitalier Universitaire de Tours, Tours, France; c Service d’Information Médicale, Epidémiologie et Economie de la Santé (SIMEES, Centre de données cliniques), Centre Hospitalier Universitaire de Tours, Tours, France; d Université de Tours, Tours, France; e Centre Régional de Pharmacovigilance et d'Information sur le Médicament, Centre Val de Loire, Centre Hospitalier Universitaire de Tours, Tours, France

**Keywords:** Recurrent sweating, night sweats, secondary hyperhidrosis, sudation, diagnostic

## Abstract

**Background:** Identification of underlying diseases is crucial for secondary hyperhidrosis management, but data are lacking to guide appropriate investigation.

**Objective:** To describe aetiologies of recurrent sweating in a hospital setting and the diagnostic performance parameters of their respective clinical/biological features.

**Patients and Methods:** We performed a monocentric evaluative study in a tertiary care centre. Patients with recurrent generalised sweating were selected *via* the Clinical Data Warehouse (CDW) by screening all electronic hospital documents from the year 2018 using a keyword-based algorithm. All in and out-patients aged ≥ 18 years having reported recurrent sweating for at least 2 weeks in 2018 were included, with a minimum one-year follow-up after symptoms’ onset.

**Results:** A total of 420 patients were included. Over 130 different aetiologies were identified; 70 patients (16.7%) remained without diagnosis. Solid organ cancers (14.3% with 13 lung cancers), haematologic malignancies (14.0% with 35 non-Hodgkin’s lymphomas) and Infectious Diseases (10.5% including 13 tuberculosis) were the most frequent diagnoses. Other aetiologies were gathered into inflammatory (16.9%) and non-inflammatory (27.6%) conditions. To distinguish non-inflammatory and undiagnosed hyperhidrosis from other causes, fever had a specificity of 94%, impaired general condition a sensitivity of 78%, and C-reactive protein (CRP) > 5.6 mg/l a positive predictive value of 0.86. Symptoms’ duration over 1 year was in favour of non-infectious and non-malignant causes (94% specificity).

**Conclusions:** We identified fever, impaired general condition, duration, and CRP as helpful orientation parameters to assess the need for complementary explorations for hyperhidrosis. The study provides a diagnostic algorithm for the investigation of recurrent sweating.

**KEY MESSAGES**
- In a hospital setting, malignancies and infections are the most frequently associated diseases, but 1/5 remain without diagnosis.
- Fever is a specific but not sensitive sign to distinguish inflammatory conditions.
- Over 1 year duration of symptoms significantly reduce the probability of malignancy or infection as the underlying diagnosis.

## Introduction

Thermoregulatory sweating is a physiological mechanism to maintain thermoregulation homoeostasis in humans. Sweating is triggered by a body temperature increase, which is sensed by peripheral and central thermoreceptors under the central control of the hypothalamus [1,2]. The hypothalamus, in turn, activates the autonomic nervous system through the efferent sympathetic pathway. Sweat is primarily produced by eccrine glands, which are widespread throughout the skin in significant numbers (1.6–4 million on the whole body) with variable density. These glands have muscarinic receptors that can be bound to acetylcholine, a neurotransmitter released from sudomotor nerves. Upon stimulation, human sweat glands can produce an average sweat rate of 1.4 l/h. This rate is regulated by body fluid volume and mechanically by skin hydration status [2].

In certain conditions, sweating rate can increase apart from exercise or elevated environmental temperature. Isolated, sweating out of those settings could be defined as primary hyperhidrosis, with a poorly understood pathophysiology, which may involve an autonomic pathway overstimulation exceeding thermoregulatory needs [3,4]. Hyperhidrosis is defined as excessive sweating causing negative impact on patients’ quality of life, and is thus a subjective symptom [5]. Secondary hyperhidrosis relies on an underlying condition. Excessive sweating has been reported in association with numerous diseases, including tuberculosis, lymphoma, and endocarditis, typically occurring at night, wetting clothes and bedsheets [6,7]. Sweating is often associated with fever as an appropriate response to body temperature increase, although there is not always body temperature elevation in secondary hyperhidrosis. In contrast to the more frequent focal presentation of primary hyperhidrosis, secondary hyperhidrosis is usually generalized to the whole body [5]. The proper diagnosis of secondary hyperhidrosis is crucial as life-threatening diseases can potentially be involved. Besides the consequences due to the underlying disorder, hyperhidrosis impacts quality of life by itself, notably involving psychosocial impairment [8,9].

To date, very limited data are available regarding the potential aetiologies and epidemiology of hyperhidrosis, although this symptom is quite common in primary care [10,11]. Studies in this field are mostly represented by literature reviews of case reports and are not able to provide the frequency of each diagnosis [6,7,12]. Only one retrospective study in an outpatient dermatology department has provided valuable clinical information about the differentiation of secondary hyperhidrosis from primary hyperhidrosis [13]. However, based on only one department, primary hyperhidrosis was predominantly represented along with few underlying diseases, and no data in hospitalised patients were available. Clinicians are thus lacking guidelines to support appropriate extensive investigation when they are confronted with generalised sweating in adults.

The aim of this study was to assess the possible aetiologies of recurrent sweating in a hospital setting and the diagnostic performance parameters of respective clinical and biological features, using one teaching hospital data warehouse.

## Patients and methods

A retrospective cohort of patients with recurrent generalised sweating was built to collect clinical and biological features from all in- and out-patients of one university hospital using the electronic medical records from a Clinical Data Warehouse for the year 2018.

Because of the absence of diagnostic criteria for recurrent sweating, we empirically based our hypothetical definition of recurrent sweating to tend towards the standard definition of prolonged fever of unknown origin, considering symptoms occurring a minimum of 2 weeks from the onset of symptoms [14].

### Data source

Data were collected using eHOP®, the Clinical Data Warehouse software of the tertiary care hospital of Tours; it integrates all electronic medical documents produced in the hospital information system [15,16]. We developed a research algorithm to detect the keywords “sweat” and its synonyms in French, including plural spelling, which were subsequently followed, within fewer than four words, by the French words for “prolonged”, “chronic”, “recurrent”, “night”, “day(s)”, “week(s)”, “month(s)”, or “year(s)”. The algorithm excluded documents where keywords were preceded by a negation within fewer than seven words.

### Studied population and data collection

Our research algorithm was performed based on the data warehouse screening among all hospital documents produced between 1 January 2018 and 31 December 2018. All adults ≥ 18 years old admitted as in-patients and out-patients in 2018 were selected. As the datamining was carried out up to December 2020, all patients had at least a one-year follow-up after the onset of symptoms. We thoroughly analysed each patient’s medical record to include all patients who reported generalized sweating episodes for at least two weeks. Initial diagnosis was defined as the imputed diagnosis for the recurrent sweating symptomatology at hospital discharge. Final diagnosis for each secondary hyperhidrosis case was the most recent imputed diagnosis provided by the referring hospital physician at the time of data collection. Other data collected from the data warehouse included: age, sex, department of hospitalisation/consultation, duration of symptoms, associated symptoms, medical history, treatments, and laboratory results.

### Pharmacovigilance database

We searched in PubMed and Google Scholar electronic databases by using combinations of the following keywords: “hyperhidrosis”, “sweating” and “drug-induced”, “drugs”, “medications”. For drugs of interest that were suspected by the attending physician, we identified all cases of “hyperhidrosis” (using preferred term according to MedDRA classification) reported to French pharmacovigilance database (FPVD) and to the WHO pharmacovigilance database (Vigilyze®) until January 2022. Cases with multiple suspected drugs were excluded. Only drugs with more than five cases in FPVD and 100 cases in Vigilyze® were labelled as reported in the respective database.

### Statistical analysis

Variations between two groups were compared using Student’s *t*-test. Diagnostic performances for the dependent variable for a binary criterion, including sensitivity, specificity, positive predictive value (PPV), and negative predictive value (NPV), were estimated using the associated contingency table [17]. Accuracy was defined as: (True positive + True negative)/total number of patients. Continuous biological variables were assessed using an ROC curve in order to establish an optimal cut-point for diagnosis. Sensitivity and specificity were assessed for different cut-points and the optimal cut-point was defined as the maximisation of Youden’s index. ROC curve confidence intervals were estimated using bootstrap simulations on the performance measures. For all calculations, statistical significance was defined as a *p*-value of < .05. Statistical analyses were performed using both R software version 3.1 through the GMRC Shiny Stat interface from Strasbourg University Hospital (2017) and GraphPad Prism version 9.1.1.

### Ethical approval

Ethical approval (no. 2020_057) was provided by the Ethics Committee in Human Research ERERC Centre Val de Loire, Tours, France (Chairperson Dr B. Birmele) on 10 July 2020. No patient consent was needed as this retrospective study was non-interventional. All patients admitted to Tours University Hospital are informed about and able to refuse the use of their anonymized data for research purposes.

## Results

### Flow chart

**Figure 1.**
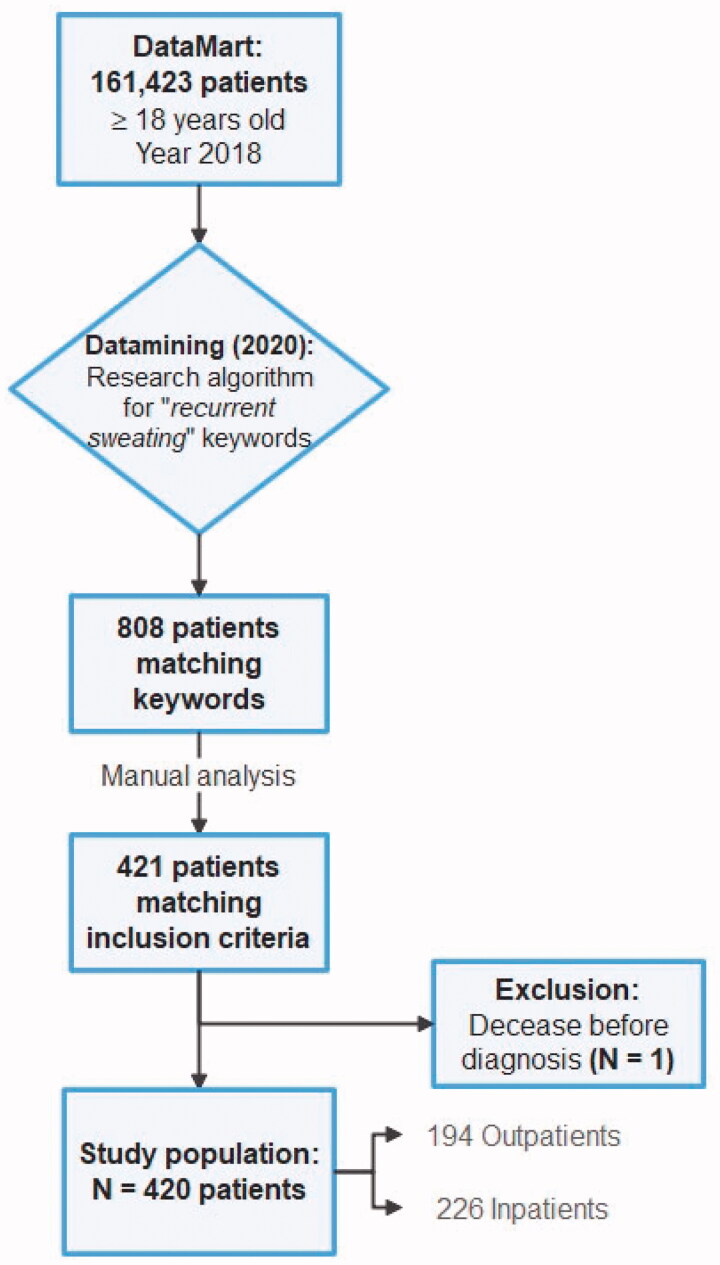
Flow chart.

Our algorithm found 808 patients matching our primary research criteria. A total of 421 patients matched inclusion criteria following our manual analysis of medical records, out of 161,423 patients from our DataMart (0.3%). One patient was excluded due to an early death before any diagnosis was made. Eventually, 420 initial and final diagnoses were collected and enabled including 420 patients (Figure 1).

### Referring departments

Recurrent sweating was reported in patients from 27 different medical departments among the same tertiary care centre during the year 2018, including both out-patients and in-patients (Appendix 1). Half of those reports were spread across only four departments: Internal Medicine, Haematology, Pneumology, and Infectious Diseases (*N* = 223/50.2%; single-patient multiple admissions being counted individually).

### Aetiological findings

Recurrent sweating occurred among 420 patients and over 130 aetiologies or groups of pathologies were identified after at least one year of follow-up (Table 1 and Appendix 2). No secondary cause was found for 70 patients (16.7%). Malignancy was the most frequent diagnosis group, including 119 patients (28.3%), half of them presenting with haematologic malignancies. Non-Hodgkin’s lymphoma (*N* = 35) was the most frequent haematologic malignancy and lung cancer (*N* = 13) was the most frequent solid organ cancer. Infectious diseases were identified for 44 patients (10.5%) and medications were involved in 33 patients (7.9%). Tuberculosis was the most frequently involved infection (*N* = 13). Drug-induced recurrent sweating included 19 different pharmacological classes (Table 2). All the patients’ medication data at the time the symptoms occurred were collected and each pharmacological class frequency was compared between patients, with no aetiological findings for their symptomatology and patients with established diagnoses as a comparison group. No statistical difference could be found between the two groups (data not shown). Systemic autoimmune and autoinflammatory diseases were identified in 24 patients (5.7%) and endocrine diseases in 22 (5.2%). Other following organs/systems involvement represented less than 5% of the diagnoses each: rheumatologic, psychiatric, neurologic, respiratory, non-oncologic haematologic, digestive and cardiovascular diseases. Non-pathologic conditions including menopause and pregnancy were also associated with recurrent sweating in 6 patients (1.4%). Surgical complications, obesity, allergies, dermatologic, ear nose throat (ENT), gynaecologic, and environmental diseases were among the rarest diagnoses and involved less than 1% of patients each. Extensive follow-up provided a diagnosis for 14 additional patients who did not have one at the end of their initial exploration. It also corrected the initial diagnosis in 13 cases.

**Table 1.** Main aetiologies of recurrent sweating.

Aetiologies		*N*	*N* (%)
Cancer (non-haematologic)	Lung carcinoma	13	60 (14.3)
	Renal carcinoma	6	
	Breast carcinoma	5	
	Cholangiocarcinoma	5	
	Pancreatic adenocarcinoma	5	
	Colorectal adenocarcinoma	4	
	Prostate adenocarcinoma	3	
	Hepatocellular carcinoma	2	
	Kaposi’s sarcoma	2	
	Leiomyosarcoma	2	
	Melanoma	2	
	Non-seminomatous mediastinal germ cell tumour	2	
	Metastasis (undetermined)	2	
Cardiovascular	High blood pressure	4	9 (2.1)
	Vasovagal syncope	2	
Digestive	Crohn’s disease	5	10 (2.4)
	Ulcerative colitis	2	
	Chronic inflammatory bowel disease (unspecified)	2	
Endocrine	Dysthyroidism	8	22 (5.2)
	Pituitary adenoma	5	
	Adrenal insufficiency	2	
	Diabetes	2	
	Ovarian insufficiency	2	
Haematologic			
Oncologic	Lymphoma	47	59 (14)
	Non-Hodgkin’s lymphoma	35	
	Hodgkin’s lymphoma	11	
	Chronic lymphocytic leukaemia	8	
	Multiple myeloma	3	
Non-Oncologic	Myelofibrosis	3	12 (2.9)
	Histiocytosis	2	
Infectious diseases	*Mycobacterium tuberculosis*	13	44 (10.5)
	Non-tuberculous mycobacteria	2	
	HIV	3	
	Cytomegalovirus	3	
	*Helicobacter pylori* gastritis	2	
	Chronic osteoarticular infection	3	
	Male urinary tract infection	2	
	Malaria	2	
	Pneumonia	2	
	Post-viral syndrome	2	
Medications	(see Table 2)		33 (7.9)
Neurologic	Peripheral neuropathy	4	13 (3.1)
	Neuropathic pain	3	
	Dysautonomia	2	
Physiologic	Menopause/perimenopause	5	6 (1.4)
Psychiatric	Depressive and/or anxiety disorder	12	17 (4)
	Withdrawal syndrome	3	
Respiratory	Obstructive sleep apnoea	5	12 (2.9)
Rheumatologic	Peripheral spondyloarthropathy	3	19 (4.5)
	Fibromyalgia	2	
	Rheumatoid arthritis	2	
	RS3PE	2	
	Arthritis (unspecified)	2	
Systemic	Vasculitis	6	24 (5.7)
(General,	Lupus	3	
Autoimmune or	Sarcoidosis	3	
Autoinflammatory)	Autoinflammatory disease (unspecified)	3	
	Still’s disease	2	
Primary hyperhidrosis / No aetiology		70	(16.7)
Others*		10	(2.3)

*N* = 420 patients (aetiologies with a frequency of 1 in 420 are not shown and are presented in Appendix 2).

*Including allergies, dermatologic, and gynaecologic diseases, ENT-related diseases, environmental and nutritional diseases, and surgical complications.

**Table 2.** Drug-induced recurrent sweating.

Pharmacological class	*N*	Main reported drugs	Pathophysiology
Corticosteroids	3	^b^	Increased sympathetic activity through glucocorticoid receptors
Immunosuppressive therapy	3	Tacrolimus^a^, Mycophenolate mofetil^b^, Leflunomide^c^	
Anti-PD-1 Ab	2	Pembrolizumab	
TNFα inhibitors	2	Adalimumab^a^, Infliximab^a^	Imbalance in cytokines network
Anti-IL-17 Ab	1	Ixekizumab	
Tyrosine kinase inhibitors	1	Sunitinib	
Chemotherapy (unspecified)	1	^b^	
Anti-androgen/GnRH agonists	3	Cyproterone acetate, Leuprorelinea + Abiraterone, Triptorelin^a^	Anti-estrogenic action
Aromatase inhibitor	2	Letrozole^a^, Exemestane^c^	Anti-estrogenic action
Estrogen receptor modulators	1	Tamoxifen^b^	Anti-estrogenic action
Hormonal contraception	1	Ethinylestradiol + Levonorgestrel^c^	
Antiretroviral	1	Emtricitabine + Rilpivirine + Tenofovir alafenamide	
Morphine	2	Fentanyl^a^	Histamine release
Cholinesterase inhibitors	1	Pyridostigmine^a^	Cholinesterase inhibition
SNRI	3	Venlafaxine^a^	Serotonergic effect on hypothalamus or spinal cord
SSRI	3	Paroxetine^a^, Fluoxetine^a^	Serotonergic effect on hypothalamus or spinal cord
GABA-R modulators	1	Etifoxine	
Antipsychotic	1	Paliperidone	
Antiepileptic	1	Lamotrigine^c^, Lacosamide	

Ab: antibodies; SNRI: Serotonin-Noradrenaline Reuptake Inhibitors; SSRI: Selective Serotonin Reuptake Inhibitors. For precise database results, see Appendix 3.

^a^Medications with a mention of sweat-inducing potential in their summary of product characteristics.

^b^Medications with no mention of sweat-inducing potential in their summary of product characteristics but with a previously reported association with sweating in scientific literature^[^12^^,^^13^^,^^20–24^]^.

^c^Medications with no mention of sweat-inducing potential in their summary of product characteristics nor in scientific literature but with reported cases in pharmacovigilance databases.

### Clinical and biological characteristics

We divided the patients into five groups: malignancies, infection, other inflammatory diseases, other non-inflammatory diseases, and the absence of aetiological findings, based on the pathophysiological mechanisms of each disease (Table 3). We provided each group with clinical and biological characteristics. If overall sex ratio was close to 1, men did more frequently present with malignancies (60%) and infections (70%) than women. Patients with malignancies were significantly older than other groups (64 [IQR 51.5–73] vs 50 [IQR 36–63] years; *p* < .01). BMI did not differ between groups. Immunodeficiency was more frequent in patients with other inflammatory diseases, notably due to immunosuppressive treatments (*N* = 17; 23.9%). Four of 15 patients with solid organ transplants remained without diagnosis. Among patients without diagnosis, six (8.6%) had a history of sleep apnoea and 25 (35.7%) were smokers. Patients with non-inflammatory diseases and those without diagnosis were less frequently hospitalised (39.7% and 34.3% vs 67.2% for malignancies, 70.5% for infections, and 63.4% for other inflammatory diseases) and were largely treated in out-patient clinics.

**Table 3.** Patients’ characteristics by group of pathologies.

	Total	Malignancy	Infection	Other inflammatory	Other non- inflammatory	No diagnosis
N	420 (100)	119 (28.3)	44 (10.5)	71 (16.9)	116 (27.6)	70 (16.7)
Sex (F/M) (N)	213/207	48/71	13/31	40/31	69/47	43/27
Age (median)	53 (39–67)	64 (51.5–73)	43 (28.5–53)	49 (33.5–66)	53 (39–64)	47 (39.5–61)
BMI (median)	24 (21–27.4)	24.3 (21.4–27.1)	22.9 (19.2–25)	23.6 (21.3–28)	25 (22.5–30.8)	23.6 (20.5–27.3)
Menopause^a^ (*N*)	111 (52.4)	36 (75)	3 (23.1)	17 (42.5)	41 (59.4)	14 (33.3)
Medical history (*N*)						
ID^b^	76 (18.1)	17 (14.3)	8 (18.2)	17 (23.9)	20 (17.2)	14 (20)
HIV	17 (4)	5 (4.2)	4 (9.1)	1 (1.4)	4 (3.4)	3 (4.3)
SOT^c^	15 (3.6)	4 (3.4)	2 (4.5)	1 (1.4)	4 (3.4)	4 (5.7)
Sleep apnoea	23 (5.5)	4 (3.4)	1 (2.3)	1 (1.4)	11 (9.5)	6 (8.6)
Smokers	119 (28.3)	28 (23.5)	13 (29.5)	17 (23.9)	36 (31)	25 (35.7)
Hospitalized	226 (53.8)	80 (67.2)	31 (70.5)	45 (63.4)	46 (39.7)	24 (34.3)
Duration (N)						
15 to 21 days	19 (4.5)	2 (1.7)	10 (22.7)	3 (4.2)	3 (2.6)	1 (1.4)
≥ 21 days to 3 months	192 (45.7)	69 (58)	21 (47.7)	30 (42.3)	47 (40.5)	25 (35.7)
≥ 3 months to 12 months	124 (29.5)	39 (32.8)	12 (27.3)	21 (29.6)	32 (27.6)	20 (28.6)
≥ 1 year	85 (20.2)	9 (7.6)	1 (2.3)	17 (23.9)	34 (29.3)	24 (34.3)
Fever (N)	87 (20.9)	26 (24.8)	22 (51.2)	28 (39.4)	7 (6)	4 (5.7)
General state impairment (N)	248 (59)	98 (82.4)	33 (75)	51 (71.8)	39 (33.6)	27 (38.6)
Symptoms’ occurrence (N)						
Night only	375 (89.3)	106 (89.1)	40 (90.1)	70 (98.6)	97 (83.6)	62 (88.6)
Night and day	42 (10)	12 (10.1)	4 (9.1)	19 (26.8)	1 (0.9)	6 (8.6)
Day only	3 (0.7)	1 (0.8)	0	0	0	2 (2.9)
Laboratory characteristics						
CRP (mg/l)	8.2 (1.3–55.6)	26 (4.9–85.2)	44.1 (6.1–86.3)	14.9 (1.3–72.4)	2.2 (0.7–8.2)	1.2 (0.7–2.2)
LDH (UI/l)	235 (187–313)	261 (206–354)	235 (170–247)	235 (191–266)	223 (180–260)	183 (159–240)
WBC^d^ (10^9^ cells/l)	8 (6–10.8)	9.1 (7–13.6)	7.9 (5.8–10.5)	8.2 (5.9–11.1)	7.4 (6–8.8)	6.6 (4.8–8.4)
Lymphocytes (10^9^ cells/l)	1.7 (1.2–2.3)	1.5 (0.9–2.2)	1.6 (1.1–2.2)	1.8 (1.2–2.3)	2 (1.5–2.4)	1.9 (1.4–2.6)
Neutrophils (10^9^ cells/l)	5 (3.3–7.2)	5.7 (3.7–8.2)	5.3 (3.3–7.1)	5.2 (3.1–8)	4.5 (3.3–6)	3.9 (2.8–5.2)
Haemoglobin (g/dl)	12.9 (11.4–13.9)	11.8 (10.7–13.2)	12.8 (11.8–13.5)	12.4 (11.3–13.9)	13.6 (12.6–14.4)	13.7 (12.3–15.1)

Data are *N* (%) or median (± IQR).

^a^Among the female population. ^b^Immunodeficiency. ^c^Solid organ transplant. ^d^White blood cells.

Symptoms occurred mostly during the night (*N* = 375; 89.3%), less frequently during both night and day (*N* = 42; 10%), and exceptionally exclusively during daytime (*N* = 3; 0.7%). Symptoms occurring during both night and day were more frequently caused by other inflammatory diseases (19 of 42). Regarding duration of symptoms, for patients who reported recurrent sweating for more than 2 weeks and less than 3 weeks, 10 of 19 had an infection. Recurrent sweating with an onset between 3 weeks and 3 months before admission was more frequently reported (*N* = 192; 45.7%). When considering patients with recurrent sweating between 3 weeks and 3 months and those between 3 months and 1 year, we found similar proportions between the 5 groups of pathologies. Patients with recurrent sweating for at least one year had a lower proportion of malignancy and infection, and a higher proportion of other inflammatory and non-inflammatory diseases, as well as absence of diagnosis.

Fever and impaired general condition were more frequent as well as higher CRP, LDH, and neutrophil counts in patients with malignancy, infection, and other inflammatory diseases compared to groups of other non-inflammatory diseases and the group without diagnosis.

### Diagnostic tests

We evaluated the diagnostic performance of fever, impaired general condition (defined by asthenia, anorexia, and/or weight loss), elevation of CRP, LDH or neutrophils for the distinction between inflammatory aetiologies and others, as well as a one-year symptom duration to distinguish non-infectious and non-malignant causes from the others (Table 4). Fever had a high specificity (94%) but a low sensitivity (33%), with a positive predictive value (PPV) of 0.87 in favour of malignancies, infection, and other inflammatory diseases as compared to non-inflammatory aetiologies. Impaired general condition had a sensitivity of 78% and a specificity of 65% in favour of the same 3 groups. To distinguish malignancies, infection, and inflammation from other causes, for a threshold of 5.6 mg/l, CRP had a high PPV of 0.86. For a threshold of 226.5 UI/l, LDH had a PPV of 0.8. And for a threshold of 5.3 × 10^9^ cells/l, neutrophils had a PPV of 0.77. A duration of symptoms greater than one year had a high specificity (94%) and a PPV of 0.88 in favour of non-infectious and non-malignant diseases.

**Table 4.** Diagnostic performance of clinical and biological parameters.

Parameter	Cut-point	PPV	NPV	Sensitivity (95% CI)	Specificity (95% CI)	AUC (95% CI)	Accuracy
In favour of malignancy, infection, and other inflammatory aetiologies (vs others)							
Fever		0.87	0.53	33	94	–	0.6
Imp. Gen. condition		0.73	0.7	78	65	–	0.72
CRP	5.6 mg/l	0.86	0.57	71.1 (65–77.2)	76.5 (68.4–85)	79.2 (74.1–84.4)	0.73
LDH	226.5 UI/l	0.8	0.39	59.8 (51.2–68.5)	63.5 (50–75.1)	65.2 (56.5–73.8)	0.61
Neutrophils	5.3 × 10^9^/l	0.77	0.43	51.2 (44.5–57.9)	70.4 (62–78.7)	61.1 (54.9–67.3)	0.58
In favour of other aetiologies and primary hyperhidrosis (vs malignancy and infection)							
≥ 1 year duration		0.88	0.46	29	94	–	0.54

For continuous variables, optimal cut-points were established using a ROC curve. Sensitivity and specificity were assessed for different cut-points and the optimal cut-point was defined as the maximisation of Youden’s index. Accuracy is defined as: (True positive + True negative)/total of patients. PPV: Positive predictive value; NPV: Negative predictive value; AUC: Area under the curve; Imp. Gen. condition: Impaired general condition.

## Discussion

When confronted to the symptomatology of recurrent sweating, the recognition of underlying diseases is a key challenge for clinicians. It may sometimes be difficult to distinguish between secondary and primary hyperhidrosis. To our knowledge, no available data on the aetiologies of recurrent sweating in hospitalised patients have been published to date. Our retrospective cohort of 420 patients is the largest set of diagnoses to date associated with recurrent sweating and the largest cohort of secondary hyperhidrosis. Walling *et al.* suggested that primary hyperhidrosis onset was classically between 14 and 25 years old, with excessive sweating of more than 6 months’ duration, involving eccrine-dense sites, bilateral and symmetric, associated with a family history, and occurring only during the day [13]. We suggest that those criteria, which were developed in an outpatient dermatology department, are not applicable in certain settings, especially in hospitalised patients. Our patients were mainly over 25 years old, and other studies have reported idiopathic night sweats among elderly patients [10]. Almost all of the patients had nocturnal sweating episodes, including those identified as having undiagnosed hyperhidrosis, which may exclude day-time occurrence as a clear-cut criterion. After proper follow-up, our undiagnosed patients may be considered to actually have primary hyperhidrosis, and the current criteria might be expanded. We also showed that a duration cut-point of one year instead of 6 months might be more appropriate to consider primary hyperhidrosis in older patients, as it better decreased the risk of missing life-threatening diseases such as malignancies and infection.

**Figure 2.**
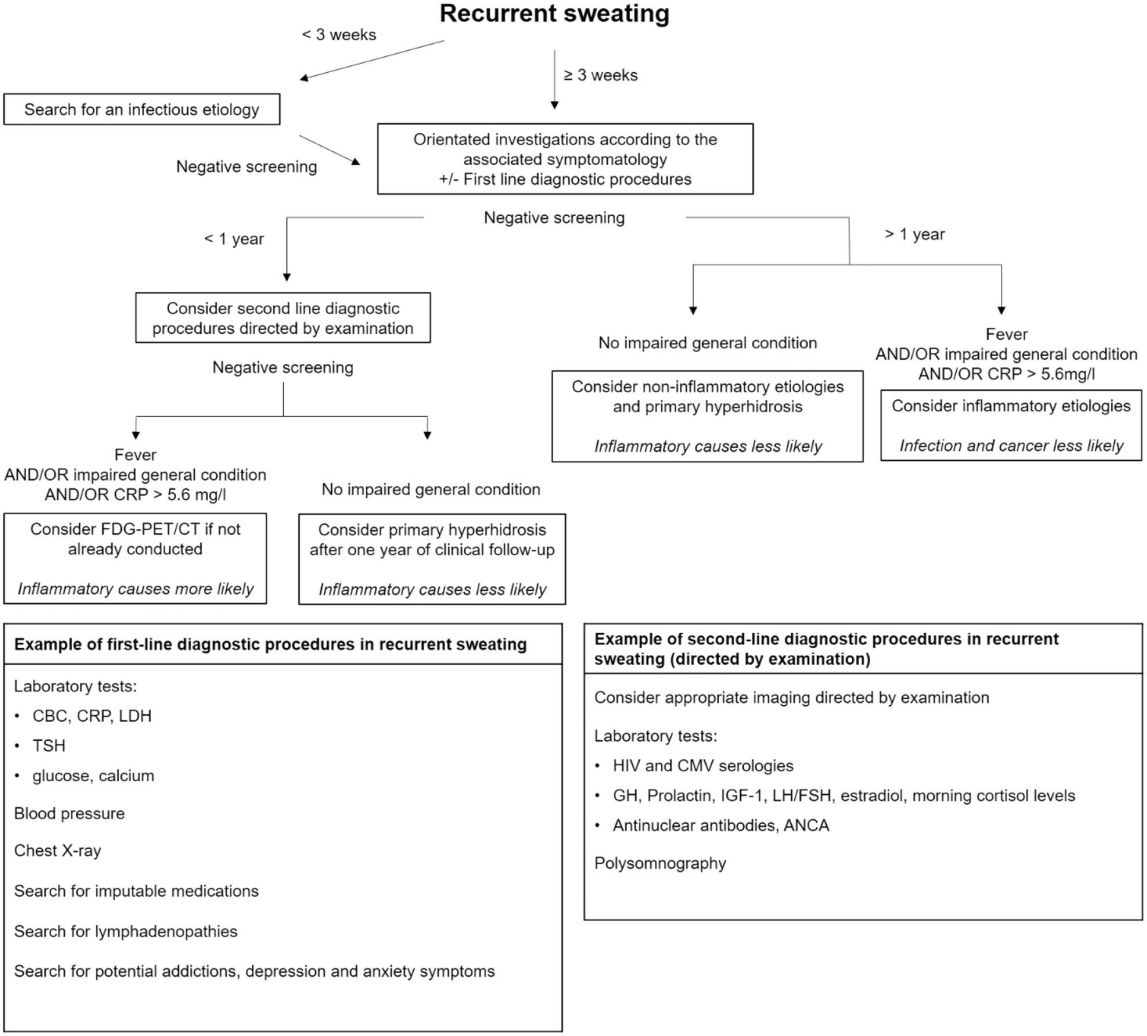
Diagnostic approach for recurrent sweating. CBC: Blood cell count; ANCA: antineutrophil cytoplasmic antibodies; TSH: thyroid-stimulating hormone: GH: growth hormone; IGF-1: Insulin-like growth factor 1; LH: Luteinizing hormone; FSH: follicle-stimulating hormone.

Taking into account our main findings, we suggest a diagnostic approach by the mean of an algorithm for the investigation of recurrent sweating (Figure 2). Duration of less than 3 weeks is mainly associated with infectious diseases. Proper investigation of recurrent sweating should be considered after at least 3 weeks’ duration. We suggest key clinical examination points and simple tests that could help orientate the investigation according to the aetiologies we report. Duration of one year or more is poorly associated with infections and malignancies, and other diagnoses should thus be considered in the absence of other signs in favour. Fever, impaired general condition, and CRP > 5.6 mg/l could be useful signs to consider inflammatory diseases. Second-line diagnostic procedures should be directed by examination. In the event of strong suspicion of malignancy or infection, FDG-PET/CT may help the investigation [18].

Several reviews detailed available therapeutic options for hyperhidrosis [19–21]. Treatment of secondary hyperhidrosis relies on the treatment of the underlying diseases. In some cases, if treatment is ineffective, unavailable, or if the symptoms are poorly tolerated, generalized hyperhidrosis could be treated using oral or transcutaneous anticholinergic drugs. Their use is however limited by their numerous adverse effects and contra-indications, especially in the elderly. Other oral alternatives include beta-blockers, clonidine, indomethacin, and calcium channel blockers, but with limited data. None have been formally approved in this indication by drug agencies. Options for the treatment of focal hyperhidrosis consist of topical antiperspirants, iontophoresis, botulinum toxin injections, and local surgical procedures including sympathectomy.

Our study’s design strength is that retrospective research using a Clinical Data Warehouse enabled us to largely include patients from multiple departments and prevented a selection bias. We aimed to provide an as complete as possible list of diagnoses associated with recurrent sweating to be considered for differential diagnosis in order not to miss the rarest aetiologies. Our study also had some limitations. Being monocentric, data came from only a single centre in France. Our results should be confirmed in other centres and might not be comparable to the aetiologies of secondary hyperhidrosis worldwide, such as tropical countries with a wider range of infectious diseases. Its retrospective design, including documents from departments which were not specialized in the management of hyperhidrosis, did not allow for detailed description of the symptomatology. Some departments may also have searched more systematically than others for the occurrence of sweating. Our centre is a tertiary university hospital and patients with rare diseases may be over-represented. Thus, although primary hyperhidrosis is usually described as more frequent than secondary hyperhidrosis, our cohort lacked younger subjects who typically present with primary hyperhidrosis. We included patients from both in and out-patient clinics in similar proportions. However, if those two groups enabled a comprehensive overview of diseases that can be faced within a hospital, they differed as hospitalised patients present with worsened conditions and should be explored accordingly. Adequate warning signs are more frequent in the event of hospitalization.

## Conclusion

We identified the main aetiologies of recurrent sweating in a university hospital setting and identified warning signs including fever, impaired general condition, alteration to or elevated CRP to help clinicians identify situations requiring extensive investigation. On the contrary, the absence of those warning signs, or a prolonged duration greater than a year, associated with a negative examination-based exploration, should not systematically be investigated by invasive or irradiating tests. Primary hyperhidrosis may have late onset and more studies are needed to confirm those findings.

## Data Availability

The data that support the findings of this study are available from Nived Collercandy, upon reasonable request. All authors had access to the data and a role in writing the manuscript.

## Appendix 1 Departments of consultation or hospitalization of patients presenting with recurrent sweating

Patients referred to multiple departments were counted in each one separately. Percentage of total is the proportion in our study population (*N* = 420).

**Table:**

Department	Total *N* (%)	Hospitalization *N*	Consultation *N*
Internal Medicine	82 (20)	48	34
Haematology	60 (14)	31	29
Pneumology	47 (11)	31	16
Infectious Diseases	46 (11)	32	14
Emergency	29 (7)	14	15
Rheumatology	25 (6)	21	4
Hepato-Gastro-Enterology	21 (5)	17	4
Nephrology & Transplantation	20 (5)	9	11
Endocrinology	19 (4)	7	12
Oncology	17 (4)	7	10
Neurology	16 (4)	8	8
Dermatology	16 (4)	8	8
Psychiatry	11 (2)	3	8
Gynaecology & Obstetrics	8 (2)	3	5
Digestive Surgery	7 (2)	4	3
Others*	36 (9)	–	–

*1%: Intensive Care, Cardiology, Neurosurgery, Orthopaedic surgery, Geriatrics, Nutrition; < 1%: ENT, Palliative care, Thoracic surgery, Nuclear Medicine, Physiatry.

## Appendix 2 Aetiologies of recurrent sweating

*N* = 420 patients.

**Table:**

Aetiologies		*N*	*N* total (%)
Allergy	Grass pollen allergy	1	(0.2)
Cancer (non-haematologic)	Lung carcinoma	13	60 (14.3)
	Metastatic	7	
	Renal carcinoma	6	
	Metastatic	4	
	Breast carcinoma	5	
	Metastatic	3	
	Cholangiocarcinoma	5	
	Metastatic	5	
	Pancreatic adenocarcinoma	5	
	Metastatic	3	
	Colorectal adenocarcinoma	4	
	Metastatic	3	
	Prostate adenocarcinoma	3	
	Metastatic	1	
	Hepatocellular carcinoma	2	
	Kaposi’s sarcoma	2	
	Leiomyosarcoma	2	
	Melanoma	2	
	Metastatic	2	
	Non-seminomatous mediastinal germ cell tumour	2	
	Adrenocortical carcinoma	1	
	Cutaneous squamous-cell carcinoma	1	
	Endometrial adenocarcinoma	1	
	Larynx carcinoma	1	
	Metastatic	1	
	Oropharyngeal carcinoma	1	
	Osteosarcoma	1	
	Pheochromocytoma	1	
	Metastasis (undetermined)	2	
Cardiovascular	High blood pressure	4	9 (2.1)
	Vasovagal syncope	2	
	Heart failure	1	
	Leriche syndrome	1	
	Orthostatic hypotension	1	
Dermatologic	Bullous pemphigoid	1	2 (0.5)
	Chronic wound	1	
Digestive	Crohn’s disease	5	10 (2.4)
	Ulcerative colitis	2	
	Chronic inflammatory bowel disease (unspecified)	2	
	Auto-immune hepatitis	1	
Endocrine	Dysthyroidism	8	22 (5.2)
	Graves’ orbitopathy	1	
	Pituitary adenoma	5	
	Growth hormone	3	
	IGF-1 + prolactin	1	
	Prolactin	1	
	Adrenal insufficiency	2	
	Diabetes	2	
	Ovarian insufficiency	2	
	Adrenal adenoma	1	
	Hypercalcemia	1	
	Thymoma	1	
ENT	Meniere’s disease	1	(0.2)
Environmental	Synthetic duvet	1	(0.2)
Gynaecologic	Endometriosis	1	2 (0.5)
	Uterine fibroid necrosis	1	
Haematologic			
Oncologic	Lymphoma	47	59 (14)
	Non-Hodgkin’s lymphoma	35	
	Diffuse large B-cell lymphoma	16	
	T-cell lymphoma	5	
	Follicular lymphoma	4	
	Mantle cell lymphoma	4	
	Waldenström’s macroglobulinemia	3	
	Marginal zone lymphoma	1	
	Hodgkin’s lymphoma	11	
	Chronic lymphocytic leukaemia	8	
	Multiple myeloma	3	
	Acute lymphoblastic leukaemia	1	
Non-Oncologic	Myelofibrosis	3	12 (2.9)
	Histiocytosis	2	
	AL Amyloidosis	1	
	Essential thrombocythemia	1	
	Hemophagocytic lymphohistiocytosis (no primary aetiological finding)	1	
	Lymphoid hyperplasia	1	
	Lymphangiomatosis	1	
	Mastocytosis	1	
	Myelodysplastic syndrome	1	
Infectious diseases	*Mycobacterium tuberculosis*	13	44 (10.5)
	Pulmonary	10	
	Lymphadenitis	5	
	Pericarditis	1	
	Spinal/bone	1	
	Erythema nodosum	1	
	Non-tuberculous mycobacteria	2	
	HIV	3	
	*Pneumocystis jirovecii*	1	
	*Mycobacterium intermediae*	1	
	Cytomegalovirus colitis	1	
	Non-compliance with treatment	1	
	Cytomegalovirus (acute infection)	2	
	Cytomegalovirus (reactivation)	1	
	Parvovirus B19 (acute infection)	1	
	*Helicobacter pylori* gastritis	2	
	Chronic osteoarticular infection	3	
	*Cutibacterium acnes*	1	
	*Staphylococcus epidermidis*	1	
	*Streptococcus mitis*	1	
	Male urinary tract infection	2	
	Endocarditis	1	
	*Staphylococcus aureus*	1	
	*Bartonella henselae* (meningitis and hepatitis)	1	
	*Coxiella urnetiid* / Q fever (acute infection)	1	
	*Plasmodium ovale*	1	
	*Plasmodium vivax*	1	
	Post-streptococcal erythema nodosum	1	
	Pneumonia	2	
	Pulmonary abscess	1	
	Cutaneous abscess	1	
	Ovarian abscess (with peritonitis)	1	
	Viral infection (undetermined)	1	
	Post-viral syndrome	2	
	Non documented infection	1	
Medications	Corticosteroids	3	33 (7.9)
	Tacrolimus	1	
	Mycophenolate mofetil	1	
	Leflunomide	1	
	Pembrolizumab	2	
	Infliximab	1	
	Adalimumab	1	
	Ixekizumab	1	
	Sunitinib	1	
	Chemotherapy (unspecified)	1	
	Cyproterone acetate	1	
	Ethinylestradiol + Levonorgestrel	1	
	Enantone + Abiraterone	1	
	Exemestane	1	
	Tamoxifen	1	
	Letrozole	1	
	Triptorelin	1	
	Emtricitabine + Rilpivirine + Tenofovir alafenamide	1	
	Morphine	2	
	Pyridostigmine	1	
	Venlafaxine	3	
	Paroxetine	2	
	Fluoxetine	1	
	Etifoxine	1	
	Paliperidone	1	
	Lamotrigine + Lacosamide	1	
Nutrition	Obesity	1	(0.2)
Neurologic	Peripheral neuropathy	4	13 (3.1)
	Neuropathic pain	3	
	Dysautonomia	2	
	Chronic polyradiculoneuritis	1	
	Occipital neuralgia	1	
	Small fibre neuropathy	1	
	Tolosa-Hunt syndrome	1	
Physiologic	Menopause/perimenopause	5	6 (1.4)
	Pregnancy	1	
Psychiatric	Depressive and/or anxiety disorder	12	17 (4)
	Depressive disorder	4	
	Anxiety disorder	10	
	Withdrawal syndrome	3	
	Burn-out	1	
	Psychogenic non-epileptic seizure	1	
Respiratory	Obstructive sleep apnoea	5	12 (2.9)
	Asthma	1	
	Chronic obstructive pulmonary disease	1	
	Cystic fibrosis	1	
	Lymphocytic interstitial pneumonia	1	
	Post-infectious bronchiolitis	1	
	Post-radiotherapy pneumonia	1	
	Pulmonary arterial hypertension	1	
Rheumatologic	Peripheral spondyloarthropathy	3	19 (4.5)
	Fibromyalgia	2	
	Rheumatoid arthritis	2	
	RS3PE	2	
	Arthritis (unspecified)	2	
	Ankylosing spondylitis	1	
	Avascular necrosis of the talus	1	
	Chondrocalcinosis	1	
	Complex regional pain syndrome	1	
	Polymyalgia rheumatica	1	
	Sciatica	1	
	Seronegative arthritis	1	
	Inflammatory arthromyalgia (unspecified)	1	
Surgical complication	Post-operative pain	1	2 (0.5)
	Inflammatory periprosthetic lymphocele (breast)	1	
Systemic	Vasculitis	6	24 (5.7)
(General,	Giant cell arteritis	1	
Autoimmune or	Granulomatosis with polyangiitis	2	
Autoinflammatory)	Microscopic polyangiitis	1	
	Polyarteritis nodosa	1	
	Not specified	1	
	Lupus	3	
	Sarcoidosis	3	
	Autoinflammatory disease (unspecified)	3	
	Still’s disease	2	
	Familial Mediterranean fever	1	
	Erythema nodosum	1	
	IgA deficiency	1	
	Non-allergic histaminic angioedema	1	
	Polyserositis (unspecified)	1	
	Polyadenitis (unspecified)	1	
	Relapsing polychondritis	1	
Primary hyperhidrosis / No aetiology		70	(16.7)

## Appendix 3 Sweat-inducing potential of drugs in medical databases

Very common (≥ 1/10); common (≥ 1/100 to < 1/10); uncommon (≥ 1/1,000 to < 1/100); rare *(*≥ 1/10,000 to *<* 1/1,000); very rare (< 1/10,000), not known (cannot be estimated from the available data).

**Table:**

Pharmacological class	Main reported drugs	Summary of product characteristics	French National PV database	Worldwide PV database (= Vigilyze)
Corticosteroids		No		
Immunosuppressive therapy	Tacrolimus Mycophenolate mofetil Leflunomide	Yes (hyperhidrosis, common) No No	1 1 **5**	80 77 **105**
Anti-PD-1 Ab	Pembrolizumab	No	0	85
TNFα inhibitors	Adalimumab Infliximab	Yes (night sweats, uncommon) Yes (hyperhidrosis, common)	**21** **64**	**4012** **1483**
Anti-IL-17 Ab	Ixekizumab	No	1	**113**
Tyrosine kinase inhibitors	Sunitinib	No	0	**181**
Chemotherapy (unspecified)				
Anti-androgen / GnRH agonists	Cyproterone acetate Leuproreline Triptorelin	No Yes (sweats, very common) Yes (hyperhidrosis, very common)	3 3 0	24 **1283** **105**
Aromatase inhibitor	Letrozole Exemestane	Yes (very common) No	**5** **6**	**267** **133**
Estrogen receptor modulators	Tamoxifen	No	4	**249**
Hormonal contraception	Ethinylestradiol + Levonorgestrel	No	**7**	**128**
Antiretroviral	Emtricitabine + Rilpivirine + Ténofovir alafenamide	No	2	24
Morphine	Fentanyl	Yes (hyperhidrosis, common)	**22**	**3166**
Cholinesterase inhibitors	Pyridostigmine	Yes (not known)	6	41
SNRI	Venlafaxine	Yes (hyperhidrosis, including night sweats, very common)	**78**	**2549**
SSRI	Paroxetine Fluoxetine	Yes (hyperhidrosis, very rare) Yes (hyperhidrosis, common)	**91** **27**	**2958** **1073**
GABA-R modulators	Etifoxine	No	1	2
Antipsychotic	Paliperidone	No	4	126
Antiepileptic	Lamotrigine Lacosamide	No No	**12** 1	**295** 39

Values in bold represent drugs that were considered sufficiently reported in their respective database (N ≥ 5 for FPVD and N ≥ 100 for Vigilyze).
